# The important role of the receptor for activated C kinase 1 (RACK1) in nasopharyngeal carcinoma progression

**DOI:** 10.1186/s12967-016-0885-x

**Published:** 2016-05-11

**Authors:** Hong Peng, Ping-Gui Gong, Jin-Bang Li, Long-Mei Cai, Le Yang, Yun-yi Liu, Kai-tai Yao, Xin Li

**Affiliations:** Department of Otolaryngology-Head and Neck Surgery, The Second People’s Hospital of Guangdong Province, Southern Medical University, Guangzhou, 510317 China; Cancer Research Institute and the Provincial Key Laboratory of Cancer Immunotherapy, Southern Medical University, Guangzhou, 510515 Guangdong China; Department of Otolaryngology-Head and Neck Surgery, Nanfang Hospital, Southern Medical University, Guangzhou, 510515 Guangdong China; Department of Pathology, The Sixth Affiliated Hospital of Guangzhou Medical University, Qingyuan, 511518 China

**Keywords:** Nasopharyngeal carcinoma, RACK1, Proliferation, Metastasis

## Abstract

**Background:**

The receptor for activated C kinase 1 (RACK1) is involved in various cancers, but its roles in nasopharyngeal carcinoma (NPC) have not yet been fully elucidated.

**Methods:**

Initially, RACK1 expression was analyzed by immunohistochemistry in NPC and normal nasopharyngeal (NP) tissues. It was also detected by qPCR and Western blot in NPC cells. Confocal microscope and immunofluorescence were performed to detect the subcellular compartmentalization of RACK1. Subsequently, after up- or down-regulating RACK1 in NPC cells, cell proliferation and migration/invasion were tested using in vitro assays including MTT, EdU, colony formation, Transwell and Boyden assays. Furthermore, several key molecules were detected by Western blot to explore underlying mechanism. Finally, clinical samples were analyzed to confirm the relationship between RACK1 expression and clinical features.

**Results:**

Receptor for activated C kinase 1 expression was much higher in NPC than NP tissues. And RACK1 was mainly located in the cytoplasm. Overexpression of RACK1 promoted NPC cell proliferation and metastasis/invasion, whereas depletion of this protein suppressed NPC cell proliferation and metastasis/invasion. Mechanistically, RACK1 deprivation obviously suppressed the activation of Akt and FAK, suggesting the PI3K/Akt/FAK pathway as one of functional mechanisms of RACK1 in NPC. Furthermore, clinical sample analysis indicated a positive correlation between in vivo expression of RACK1 with lymph node invasion and clinical stage of NPC.

**Conclusion:**

Our results demonstrate that RACK1 protein plays an important role in NPC development and progression. The upregulation of RACK1 can promote the proliferation and invasion of NPC by regulating the PI3K/Akt/FAK signal pathway. Thus, this study contributes to the discovery of a potential therapeutic target for NPC.

**Electronic supplementary material:**

The online version of this article (doi:10.1186/s12967-016-0885-x) contains supplementary material, which is available to authorized users.

## Background

Nasopharyngeal carcinoma (NPC) is an epithelial malignancy with high incidence rate in Chinese and other Southeast Asians. Most NPC patients cannot be timely diagnosed in early stage due to non-specific symptoms and deep location of lateral nasopharyngeal recess [1, 2]. Although chemoradiation therapy has recently improved the 5 year survival rate of NPC patients, clinical NPC prognosis is generally poor due to recurrence and metastasis [3]. Therefore, it is urgent to discover valuable novel biomarkers for NPC early diagnosis, prognostic evaluation, and molecularly targeted therapy.

Receptor for activated protein kinase C (RACK1) is an adaptor protein with seven WD40 motifs, 36 kDa, mainly allocated in cytosol and cytoplasmic membrane, providing a scaffold for multiple protein–protein interactions, thus involving diverse signaling pathways. RACK1 can regulate many cellular actions including cell growth, differentiation, adhesion, migration and immunity [4–8]. Recently, researchers have found the high expression of RACK1 in a multitude of tumors such as melanomas, breast cancer, colorectal cancer, pulmonary adenocarcinoma, hepatoma, esophageal squamous cell carcinoma, and oral squamous cell carcinomas. With regard to its roles in tumorigenesis, RACK1 mainly contributes to tumor growth, invasion and metastasis [9–15]. However, some contradictory results exist in its oncogenic property; this protein can exert a negative control on Src activity [16] and an inhibitory effect on Ki-Ras-mediated morphological cell transformation [17]. It is also reported to be down-regulated in gastric cancer, acting as a tumor suppressor negatively regulating the Wnt signaling [18]. Interestingly, more and more studies indicated RACK1 is interacted with some proteins (such as, ZEBRA, LMP1 and A73) encoded by Epstein–Barr virus (EBV) which has been implicated in the molecular abnormalities leading to NPC [19–22], implying that RACK1 probably impacts N NPC development and progression. Therefore, the roles of RACK1 in NPC are deserved to be explored.

In the present study, we have examined the mRNA and protein expression levels of RACK1 in NPC cell lines and tissue samples, explored the in vitro effects of RACK1 on cell proliferation and invasion/metastasis, disclosed the underlying molecular mechanism, and further evaluated the clinical relevance of RACK1 to NPC. This is the first to investigate the roles of RACK1 in NPC.

## Methods

### Tissue samples collection

NPC samples and non-cancerous nasopharyngeal samples (NP) were collected from the First Affiliated Hospital of Southern Medical University, Nanfang Hospital of Guangzhou City, China between January 2012 and July 2014. All tissue specimens were obtained at the time of nasal endoscopy diagnosis before any anti-tumor therapy. All biopsy samples had been confirmed by pathological diagnosis. TNM staging was performed according to the 7th Edition of the AJCC/UICC Cancer Staging Manual (The 2009 NPC staging system of the WHO). Prior consents were obtained from the patients before using these clinical materials for research. The study was also approved by the Ethics Committee of Nanfang hospital. Each tissue was fixed in buffered formalin for 48 h, embedded in paraffin and sectioned, stored at 4 °C before immunohistochemical staining.

### Immunohistochemistry

Paraffin sections (5 μm) from samples of 58 NPC and 37 nasopharyngeal specimens were deparaffinized in 100 % xylene and re-hydrated in descending ethanol series (100, 90, 80, 70 % ethanol) and water according to standard protocols. Heat-induced antigen retrieval was performed in 10 mM citrate buffer for 2 min at 100 °C. Endogenous peroxidase activity and non-specific antigen were blocked with 3 % hydrogen peroxide and serum, followed by incubation with mouse anti-human RACK1 antibody (1:500 dilution, BD Biosciences, SanJose, CA, USA) for overnight at 4 °C. After washing, the sections were incubated with biotin-labeled anti-mouse IgM antibody (Bosder Inc, Wuhan, China) for 30 min at room temperature, and subsequently were incubated with SABC (Bosder Inc, Wuhan, China) for 30 min at room temperature. The peroxidase reaction was developed using 3, 3-diaminobenzidine chromogen solution in DAB buffer substrate. Sections were visualized with DAB and counterstained with hematoxylin, mounted in neutral gum, and analyzed using a bright field microscope. All sections were scored by two independent pathologists, and the staining index was calculated as the product of the staining intensity (1, no staining; 2, weak staining; 3, moderate staining; 4, strong staining) and the proportion of positive cells (1, <10 %; 2, 10–35 %; 3, 35–70 %; 4, >70 %).

### Cell culture

All cells were graces from Cancer Research Institute of Southern Medical University. Human NPC cell lines (5–8F, CNE-1, CNE-2, 6–10B, SUNE-1, and HONE1) were cultured in RPMI-1640 (HyClone) medium supplemented with 10 % fetal bovine serum (Gibco). The immortalized nasopharyngeal epithelial cell line NP69 was grown in keratinocyte/serum-free medium (Invitrogen) supplemented with bovine pituitary extract. Cells were incubated at 37 °C in a humidified incubator of 5 % CO_2_.

### RNA extraction, cDNA synthesis and quantitative PCR

Total RNA was extracted using TRIzol reagent (Invitrogen) according to the manufacturer’s instructions. 1 μg total RNA was reverse-transcibed to Single-strand cDNA using primeScript^®^ RT reagent kit (TaKaRa, Dalian, China) according to the manufacturer’s instructions. Real-time PCR was performed using SYBR Premix Ex Taq (TaKaRa, Dalian, China) with an Mx3005P real-time PCR system (Stratagene, La Jolla, CA, USA). The following primers were used for RACK1: 5′-AGCAGCAACCCTATCATCGTC-3′ (forward) and 5′-TGAGATCCCATAACATGGCCT-3′ (reverse). Human GAPDH was used as the normalization control, primers for GAPDH were as follows: 5′-CATGGGTGTGAACCATGAGA-3′ (forward) and 5′-GTCTTCTGGGTGGCAGTGAT-3′ (reverse). Reactions containing no template or primer were used as negative controls. Temperature cycling parameters for RACK1 and GAPDH were with an initial denaturing at 95 °C for 30 s, 45 cycles of denaturing at 95 °C for 10 s, and annealing at 60 °C for 20 s. The relative expression levels were calculated by the 2-ΔΔCT Method.

### Western blot analysis

Cells were lysed by RIPA buffer (Fdbio science Inc, Hangzhou, China) with protease inhibitor (Fdbio science Inc, China) and phosphatase inhibitor (KeyGEN, Nanjing, Jiangsu, China) on ice for 30 min. The protein concentrations were determined by the BCA method (KeyGEN, Nanjing, Jiangsu, China). Protein was diluted by sample loading buffer (4:1) and boiled at 95 °C for 5 min. 30 μg of total proteins per lane were subjected to electrophoresis on a 12 % SDS-PAGE gel and transferred to PVDF membranes (Millipore, Billerica, MA, USA). The membranes were blocked with TBST containing 5 % BSA for 1 h followed by overnight incubation at 4 °C with the primary antibodies for RACK1 (1:5000 dilution, BD Biosciences, SanJose, CA, USA), GAPDH (1:2000 dilution, Bioworld Technology, MN,US), phosphor-Akt (Ser473) and Akt (1:1000 dilution, Cell Signaling Technology, MA, USA), anti-FAK (phosphor Y397) and FAK (1:1000 dilution, Abcam, Cambridge, UK) respectively. After washing with TBST, the membranes were incubated with the secondary antibody goat anti mouse IgM (1:5000 dilution, Bosder Inc, Wuhan, China) or goat anti-mouse IgG or goat anti- rabbit IgG (1:5000 dilution, Bosder inc, China) for 1 h at room temperature. The signals were visualized by an enhanced chemiluminescence Western blot kit (Millipore, USA), quantified by ChemiDocTM CRS Molecular Imager (Bio-Rad, Amersham, USA). Protein was normalized with GAPDH.

### Confocal Microscope

Paraffin section (5 μm) were prepared as the above immunohistochemistry procedure, after blocked with 10 % normal goat serum for 30 min at room temperature, the sections were incubated with mouse anti-human RACK1 antibody (1:200 dilution, BD Biosciences, SanJose, CA, USA) and rabbit anti-human claudin-1 antibody (1:200 dilution, Thermo Fisher, USA) together for overnight at 4 °C. After washing, The secondary antibody DyLight 488 goat anti-mouse IgG (1:200 dilution, Abbkine, CA, USA) and DyLight 594 goat anti-rabbit IgG (1:200 dilution, Abbkine, CA, USA) were used together for 1 h at room temperature, DAPI was used to stain the cell nuclei at a concentration of 1 µg/ml. The sections were mounted using anti-fade mounting medium. Images were captured at 600× magnification with OLYMPUS Confocal Laser Scanning Microscope, and analysed with FV10-ASW1.7 viewer software (Olympus America Inc., USA). Investigated wavelength: 473 nm (green); red (559 nm); 405 nm (blue).

### Immunofluorescence assays

Cells were cultured on coverslips overnight (70 % confluence), fixed with 4 % formaldehyde in PBS for 15 min at room temperature, and then permeabilized with 0.5 % Triton-X-100 in PBS for 10 min. Subsequently, cells were blocked for nonspecific binding with 10 % normal goat serum in PBS at RT for 30 min, incubated with RACK1-specific antibody (1:500 dilution, BD Biosciences, USA) at 4 °C overnight. The cells were incubated with DyLight 488 goat anti-mouse IgG (1:100 dilution, Abbkine, CA, USA) at 37 °C for 1 h. The coverslips were mounted on slides using anti-fade mounting medium with DAPI. Immunofluorescence images were acquired on Nikon ECLIP SE 80i microscope (Nikon, Tokyo, Japan). The excitation/emission wavelengths: green/EX: 450–490 nm, EM: 520 nm; blue/EX: 340–380 nm, EM:435–485 nm.

### Plasmids and small interfering RNA transfection

GFP-RACK1 and GFP-vector were purchased from Addgene Corporation, America. The following siRNAs designed based on the human RACK1 cDNA (GenBankTM accession number GNB2L1) were synthesized by Ribo bioscience Inc, Guangzhou, China. The sequences of RACK1 siRNA are si1, CAGGGATGAGACCAACTAT and si2, CAAACACCTTTACACGCTA. After optimization experiments regarding to the si-RNA concentrations, NPC cells were transfected with 100 nM RACK1 siRNAs or 100 nM negative control siRNA using the Lipofectamine 2000 (Invitrogen) according to the manufacturer’s directions. All Western blot and functional studies were carried out in 48 h after transfection.

### Cell proliferation assay

Cell proliferation was assessed using MTT kit. The cells were seeded on 96-well microplates at a density of 3000 cells per well. At 1–3 days, the cells were incubated with 20 μl of MTT labeling reagent (0.5 mg/ml, Sigma-Aldrich) for 4 h, followed by the addition of 100 μl of dimethylsulfoxide (Sigma-Aldrich) into each well. The plates were kept in darkroom for 15 min and OD value was measured with BioTek ELx800 microplate photometer (BioTek ELx800, SN 21 18 05, US). Investigated wavelength: 570 nm, reference wavelength: 690 nm.

### Colony formation assay

5–8F and CNE1 cells were transfected with GFP-RACK1 plasmid or control plasmid/siRNA targeting RACK1 or unspecific srambled siRNA respectively for 24 h, and then seeded in six-well plates at a density of 500 cells per well. After cultured for 7 or 12 days, colonies were fixed with 4 % paraformaldehyde solution, stained with hematoxylin, and counted under an inverted microscope (Nikon, Tokyo, Japan).

### 5-Ethynyl-2′-deoxyuridine (EdU) incorporation assay

Cells were incubated with EdU (final concentration, 10 μM) for 2 h and analyzed using a Click-iT^®^ EdU Alexa Fluor^®^ Imaging Kit (Ribo bioscience Inc, Guangzhou, China) according to the manufacturer’s instructions. Images were captured using a Nikon ECLIP SE 80i microscope (Nikon, Tokyo, Japan) and the percentage of EdU-positive cells was measured.

### Migration and invasion assay

Cell migration assay was carried out using a transwell chamber (8 µm pore size; Corning, NY, USA). For cell invasion assay, polycarbonate membrane filters of transwell chamber need coated with Matrigel (BD Biosciences, Bedford, MA, USA). Briefly, the cells were trypsinized and suspended at a final concentration of 10^6^ cells/ml in serum-free media. The cell suspension (100 µl) was loaded into the upper wells and 10 %FBS containing media was used as a chemotatic factors in the lower wells. The plate was incubated at 37 °C for 16–20 h. After removing the cells remaining in the upper chamber, the cells migrated through the membrane were fixed in ethanol and stained using hematoxylin or Giemsa stain, and then photographed using an inverted microscope (Nikon, Tokyo, Japan). The number of migrating or invading cells was quantified by counting the cells in five random high-power fields and calculating the mean number of cells. All assays were repeated independently at least for three times.

### Statistical analysis

All statistical analyses were performed with SPSS 20.0 software. Student T test was performed for two groups. One-way ANOVA and Dunnett’s multiple comparison test was used for multiple groups. Two-way ANOVA test was conducted to analysis two factors data. All date are presented as the mean ± sem for at least three independent experiments. *P* Values were two-sided and less than 0.05 were considered statistically significant.

## Results

### The RACK1 expression in NPC cells and clinical tissues

**Fig. 1:**
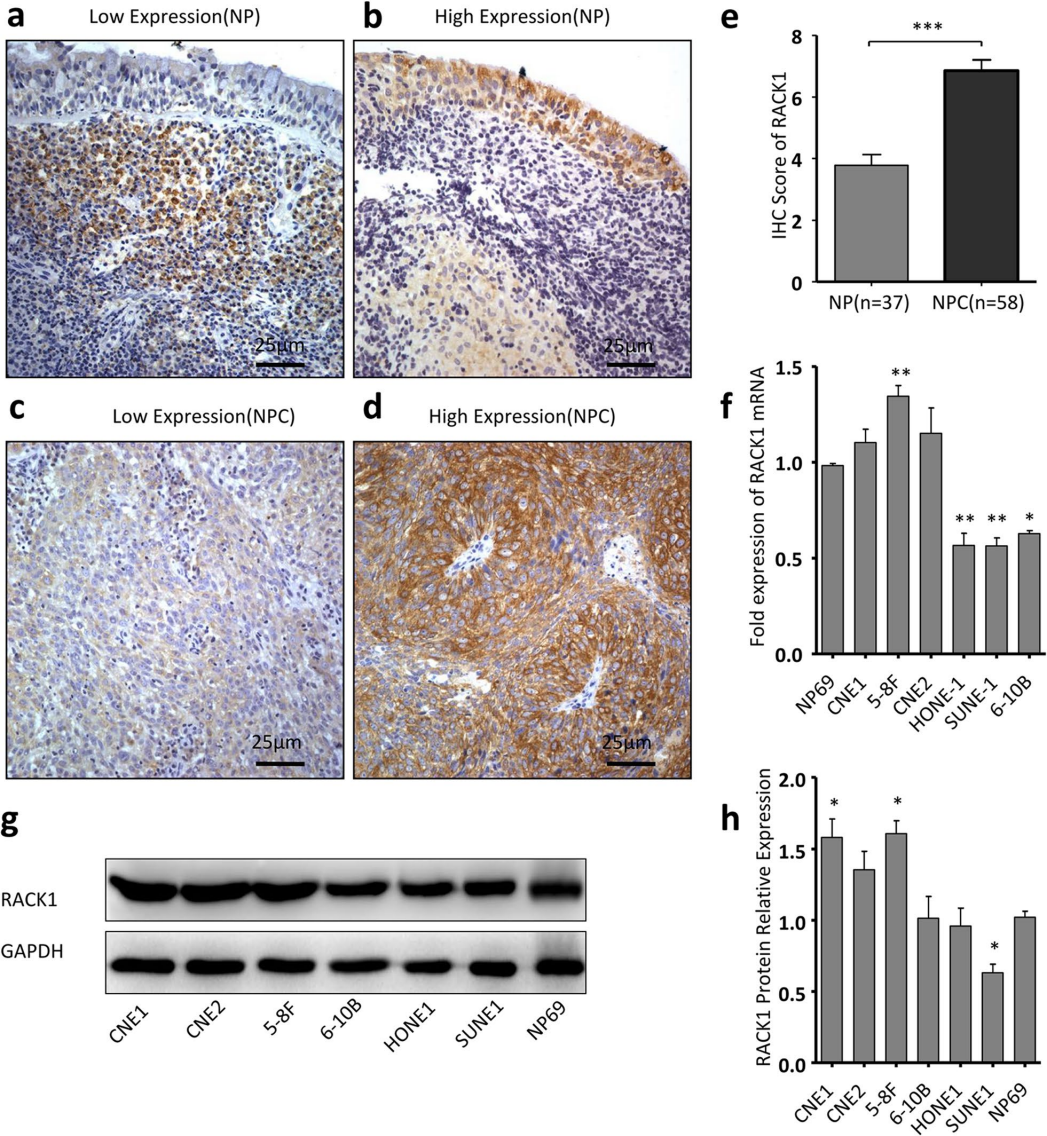
The expression of RACK1 in NPC tissues and cells. **a**–**d** The expression of RACK1 was evaluated by immunohistochemistry in NPC and NP tissues. Original magnification, ×400; *scale bar* 25 μm. **e** The histogram shows the difference of RACK1 expression (IHC score) in NPC tissues and NP tissues. Student’s *t* test. **f** The relative expression levels of RACK1 mRNA in NPC cells and NP69 determined by qRT-PCR, quantified by relating to GAPDH. One-way ANOVA and Dunnett’s multiple comparison tests. **g**, **h** RACK1 protein expressions in NPC cells and NP69 were measured by Western blot. One-way ANOVA and Dunnett’s multiple comparison test. Mean ± sem, N = 3, *P < 0.01, **P < 0.01, ***P < 0.001

To evaluate the roles of RACK1 in NPC, we initially detected the protein expression level of RACK1 in 58 paraffin-embedded NPC samples and 37 non-cancerous nasopharyngeal (NP) samples using immunohistochemical staining. Figure 1a–d showed the representative images of RACK1 expression in NPC and NP tissues. RACK1 protein was detectable in 98 % (57/58) of NPC samples and in 86 % (32/37) of NP samples. Notably, RACK1 protein expression was considerably higher in NPC samples than NP samples (P < 0.001) (Fig. 1e). 76 % (44/58) of NPC samples showed high expression level of RACK1, while only 30 % (11/37) of NP samples showed a relatively high expression level of RACK1. We then performed IF staining to define the subcellular localization of RACK1 protein in NPC tissues. Tight junction protein claudin-1 was used as a cell membrane marker (red), nuclei were stained with DAPI (blue). Confocal microscopy examinations showed that the positive staining of RACK1 (green) was observed in the cytoplasm (Additional file 1: Figure S1). These data imply that RACK1 probably plays its roles in NPC through protein–protein interaction in the cytoplasm. Moreover, we investigated RACK1 expression in NPC cell lines and immortalized nasopharyngeal epithelial cell NP69. The results showed compared to NP69, the level of RACK1 mRNA was not significantly increased in NPC cells, even a little decreased in some NPC cells (Fig. 1f). But highly invasive NPC cells (5-8F, CNE2) showed higher expression levels of RACK1 protein than relatively low malignant NPC cells (SUNE1, 6–10B) and NP69 (Fig. 1g, h). Immunofluorescence images showed the similar localization of RACK1 in NPC cells to tissue samples Additional file 1: Figure S1). These results collectively suggest that RACK1 is associated with NPC progression.

### The effect of RACK1 on NPC proliferation

**Fig. 2:**
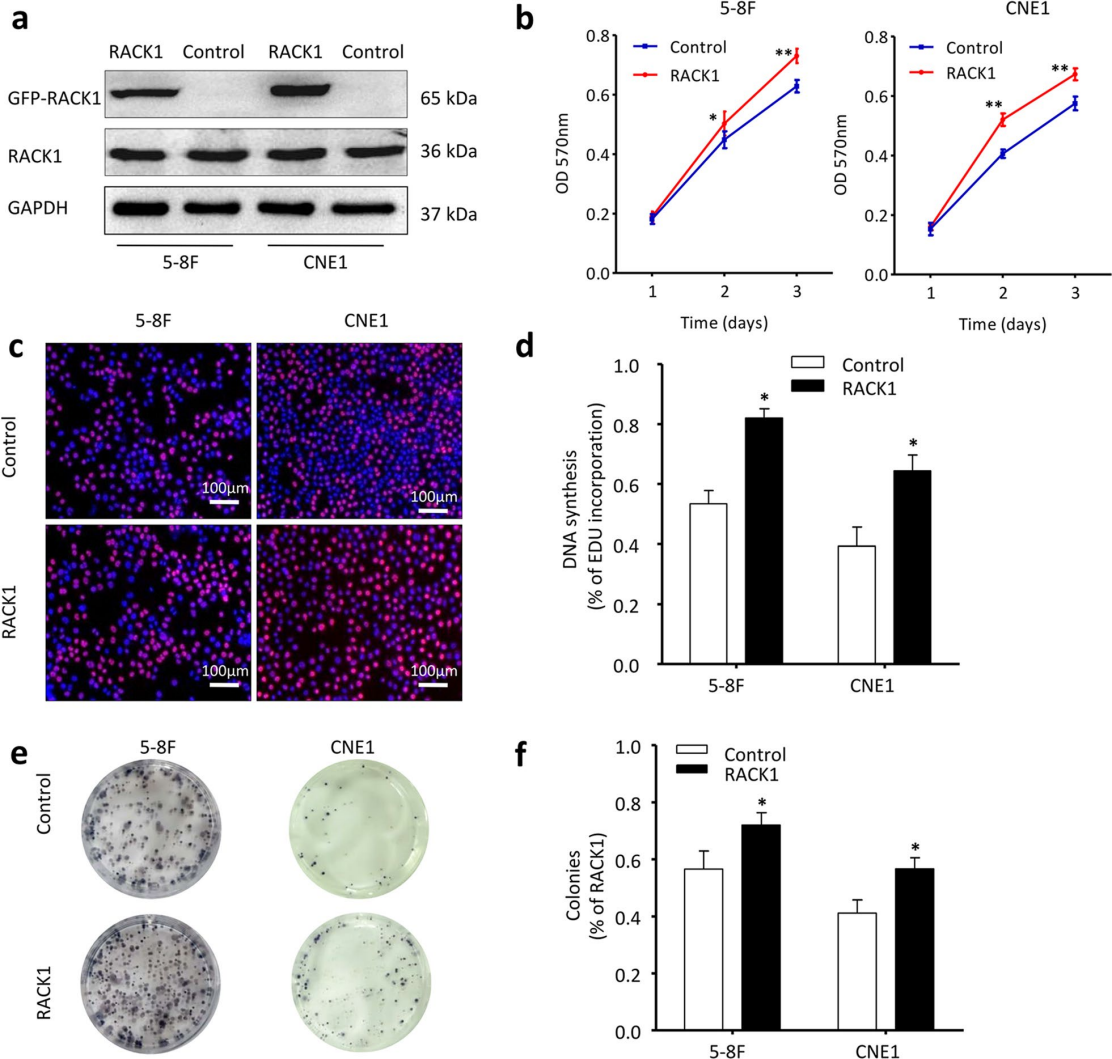
RACK1 propels NPC cells proliferation. **a** 5–8F and CNE1 NPC cells were transfected with GFP-RACK1 plasmid or control plasmid, and then analyzed by western blot. GAPDH served as the internal control. **b** MTT assays were conducted to detect the cell growth in indicated NPC cells. Two-way ANOVA test. **c**, **d** The cell proliferation status in NPC cells was also tested by EdU assay. Original magnification, ×100; *scale bar* 100 μm. Student’s t-test. **e**, **f** Besides, the clone formation ability of NPC cells was compared after transfected with RACK1 plasmid or control plasmid. Student’s t-test. Mean ± sem, N = 3, *P < 0.05, **P < 0.01, ***P < 0.001

**Fig. 3:**
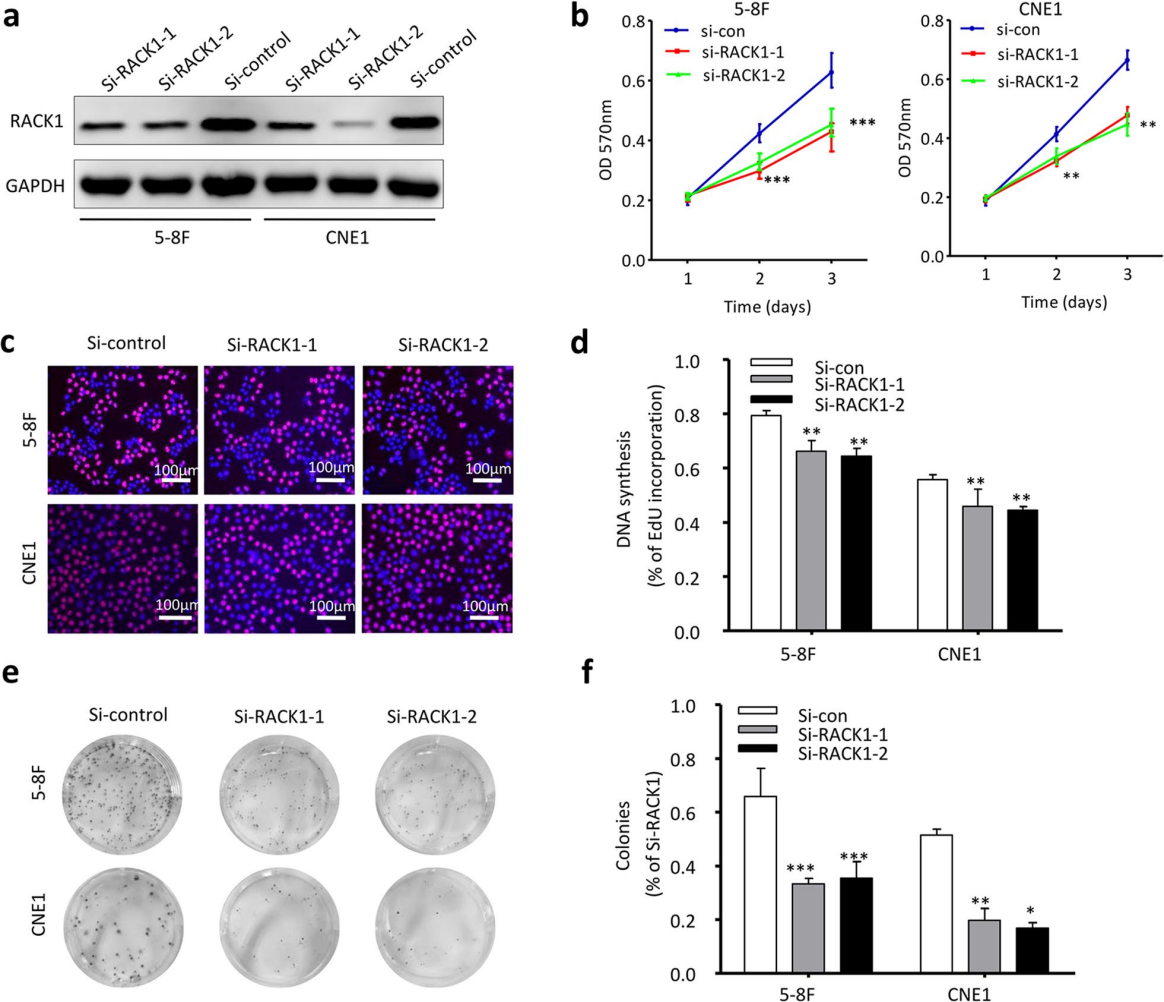
Down-regulated RACK1 attenuates cells proliferation. **a** After NPC cells transfected with RACK1 siRNA or si-control, the expression of RACK1 was detected by western blot. **b** MTT assay was applied to measure the cell growth. Two-way ANOVA test. **c**, **d** Beside, EdU assay was used to test the proliferation of NPC cells. Original magnification, ×100; *scale bar* 100 μm. Student’s t-test. **e**, **f** Plate clone formation assay also showed the difference of NPC cells proliferation. The data were shown as the mean ± sem Student’s t-test. *P < 0.05, **P < 0.01, ***P < 0.001

To investigate the effect of RACK1 on NPC tumorigenesis and progression, two NPC cells (5–8F and CNE1) were selected to be transfected with RACK1 or control plasmid. The specific RACK1 plasmid with a GFP tag, expressing a 65 kDa GFP-RACK1 fusion protein (Additional file 2: Figure S2), was used to indicate the overexpression level of exogenous RACK1 because endogenous RACK1 had already expressed in these NPC cells (Fig. 2a). Green fluorescence displayed that RACK1 protein was also more specifically localized in the cytoplasm of RACK1-transfected cells than control cells (Additional file 2: Figure S2). After plasmid transfection, MTT assays, colony formation assays and EdU assays were carried out to determine the effect of RACK1 on cell viability and proliferation ability. Overexpressed RACK1 appeared to increase cell growth (Fig. 2b), the percentage of EdU-positive cell (Fig. 2c, d), and colony formation (Fig. 2e, f) of NPC cells compared with control cells. Although the influence of RACK1 overexpression on NPC cell growth is not obvious, it has a statistical significance. Maybe as the native expression of RACK1 in NPC cell is enough, so that the results of overexpression of RACK1 are not so obvious. Subsequently, NPC cells (5–8F and CNE1) were transfected with si-RACK1 or scrambled siRNA to knock down RACK1. Relative to scrambled control siRNA, RACK1 protein expression was obviously inhibited in 5–8F and CNE1 cells treated with RACK1-siRNA (Fig. 3a). Notably, after transfection with si-RACK1, 5–8F and CNE1 cells displayed cell growth inhibition (Fig. 3b), the percentage of EdU positive cells reduced (Fig. 3c, d) as well as fewer and smaller colonies (Fig. 3e, f) compared with scrambled control. Taken together, these data indicate the promotion of cell proliferation by RACK1 in NPC.

### The effect of RACK1 on NPC migration and invasion

**Fig. 4:**
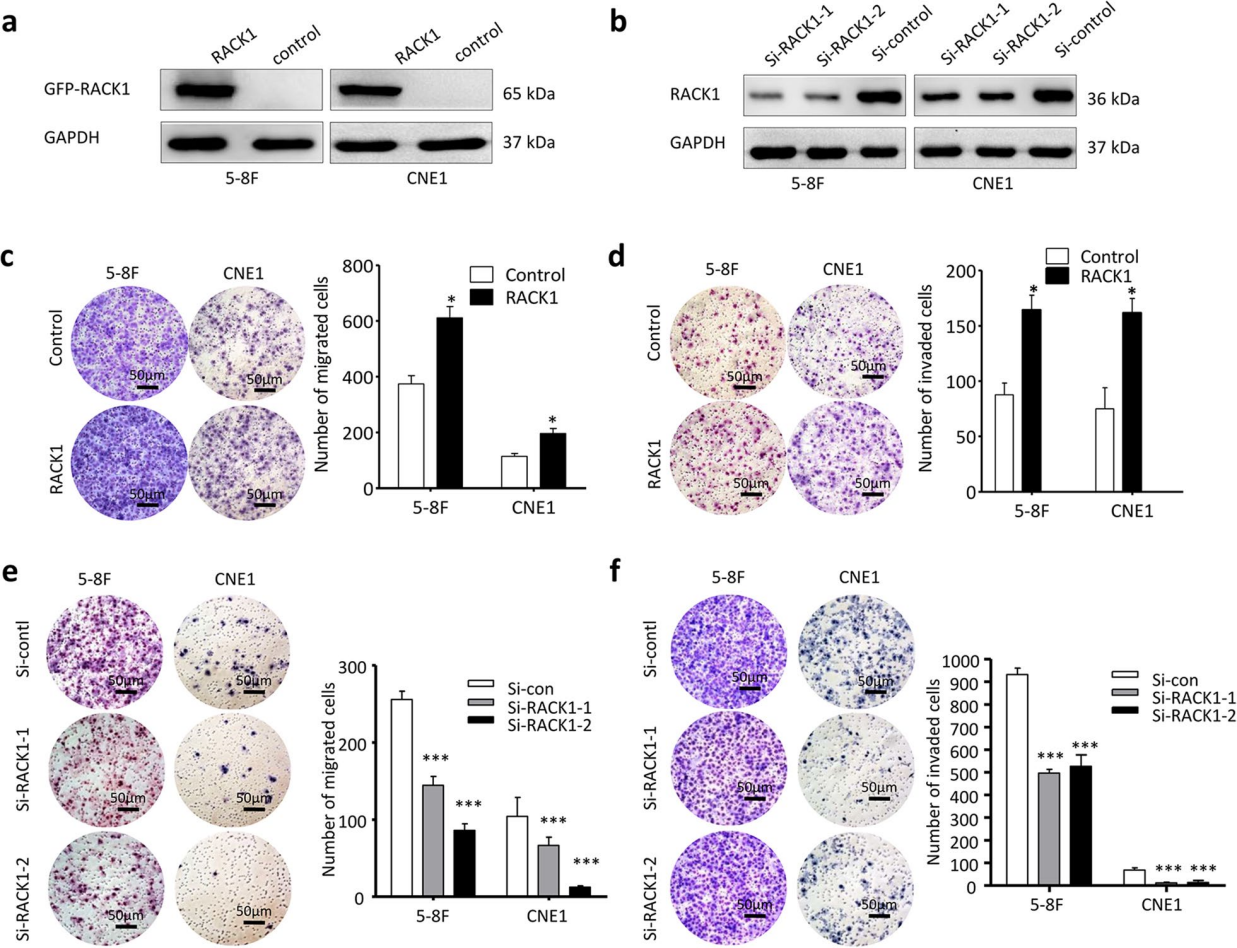
The effect of RACK1 on NPC cell migration and invasion. **a**, **b** western blots showed the successfully overexpression or knockdown of RACK1 in NPC cells. The migration and invasion of NPC cells were tested by Transwell (**c**) and Boyden assays (**d**) respectively after NPC cells were transfected with RACK1 or control plasmid. In addition, after NPC cells were treated by si-RACK1 or si-control, Transwell (**e**) and Boyden assays (**f**) were also performed to detect the migration and invasion ability of NPC cells. Original magnification, ×200; *scale bar* 50 μm. Student’s t-test, mean ± sem, N = 3. *P < 0.05, **P < 0.01, ***P < 0.001

Furthermore, the effect of RACK1 on NPC cell migration and invasion was determined using Transwell assays and Boyden assays. First, western blots were conducted to test the successfully overexpression or knockdown of RACK1 in NPC cells (Fig. 4a, b). As shown in (Fig. 4c), overexpressed RACK1 stimulated cell migration in NPC cells (5–8F and CNE1). The cell invasion abilities were also significantly increased as assessed using modified Boyden assays (Fig. 4d). Next, the migration and invasion phenotypes were compared between NPC cells treated with si-RACK1 and control cells. Similarly, down-regulation of RACK1 inhibited the migration and invasion of NPC cells (5–8F and CNE1) (Fig. 4e, f). Thus, these data demonstrate that RACK1 also enhances NPC cell migration and invasion.

### The mechanism underlying RACK1’s functions in NPC

**Fig. 5:**
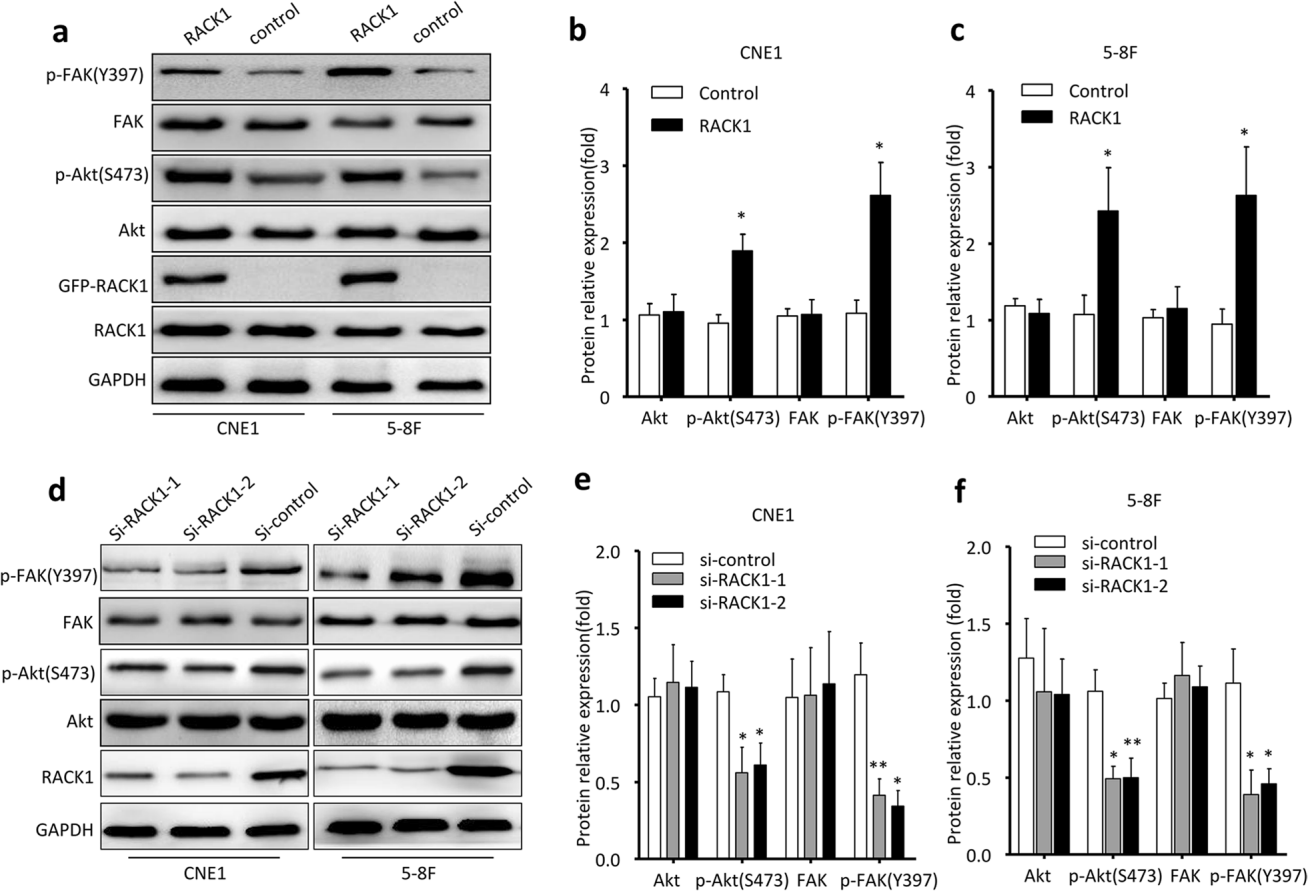
The expression of key members of PI3 K/Akt pathway in NPC cells. **a** Western blot showed the expression levels of p-Akt(S473)/Akt and p-FAK(Y397)/FAK in NPC cells transducted by GFP-RACK1 plasmid or GFP-Tag control plasmid. **d** The expression of p-Akt(S473)/Akt and p-FAK(Y397)/FAK in 5–8F and CNE1 cells was measured by western blot after transfected with RACK1 siRNA or unspecific scrambled siRNA. GAPDH served as the internal control. **b**, **c**, **e**, **f** Densitometry quantifications were performed for three independent experiments. The data were shown as the mean ± sem Student’s t-test, (*P < 0.05, **P < 0.01)

It is in NPC carcinogenesis that PI3K/Akt signal pathway has been proved to be highly implicated. Several studies have reported that RACK1 can regulate the activation of PI3K/Akt and FAK [23–30]. To determine the possible mechanism involved in the regulation of cell proliferation and migration/invasion by RACK1, we analyzed the protein levels of p-Akt(S473)/Akt and p-FAK(Y397)/FAK by western blot after overexpression or knockdown of RACK1 in NPC cells. The in vitro results revealed that RACK1 was able to activate Akt and FAK (Fig. 5a–c) and the endogenous p-Akt (S473) and p-FAK (Y397) levels were remarkably reduced in RACK1 deprived NPC cells (Fig. 5d–f). In addition, p-Akt (S473) and p-FAK (Y397) levels were restored upon the re-expression of RACK1 (Additional file 2: Figure S2). These data suggest that the PI3K/Akt/FAK pathway may be one of the regulatory mechanisms involved in RACK1’s functions in NPC. Epithelial-mesenchymal transition (EMT) is closely implicated in carcinogenesis, so the expression status of some EMT associated genes (vimentin, E-cadherin and N-cadherin) was also detected after RACK1 overexpression or deprivation. However, no significant changes were observed (Additional file 2: Figure S2), suggesting RACK1 may not participate in EMT in NPC.

### Relationship between RACK1 expression and clinicopathological variables in NPC patients

To further support our above findings, 58 NPC patients were recruited for in vivo evaluating the correlation between RACK1 protein expression level and clinicopathologic features. We did not find a significant association of RACK1 expression with patients’ age, sex, and tumor size, but we observed significant correlations of RACK1 expression with lymph node invasion and clinical stage (Table 1). These data provide further evidence that RACK1, correlating with advanced NPC, plays an important role in NPC progression.

**Table 1:** Correlation between RACK1 protein expression and nasopharyngeal carcinoma clinicopathologic features in 58 patients

Characteristics	n	RACK1 expression levels		P
		Low expression (n = 14)	High expression (n = 44)	
Gender				0.3114
Female	17	6	11	
Male	41	8	33	
Age				0.3495
>50	19	3	16	
≤50	39	11	28	
Tumor stage				0.7591
T_1_, T_2_	34	9	25	
T_3_, T_4_	24	5	19	
Lymph				*0.0471*
N0–N1	19	8	11	
N2–N3	39	6	33	
Metastasis				0.6909
Yes	10	3	7	
No	48	11	37	
Clinic stage				*0.0433*
I–II	16	7	9	
III–IV	42	7	35	

Italics mean P < 0.05, it has a statistical significance

## Discussion

A large number of studies reveal that RACK1 is associated with various signaling molecules involving the regulation of various cellular functions [4, 6–8]. Aberrant expression of RACK1 has been reported in numerous cancers [9–13]—particularly several squamous cell carcinomas such as esophageal squamous cell carcinoma (ESCC), oral squamous cell carcinoma (OSCC) and gastric cancer [14, 15, 18]. To our knowledge, the roles of RACK1 expression in NPC carcinogenesis and progression have rarely been reported. In the present study, using Western blotting and immunohistochemical analyses, we first found that RACK1 protein was expressed at a relatively higher level in NPC samples. As a scaffolding protein, RACK1 has been found to be more likely to bind to various signaling proteins in cytoplasm and several cell surface receptors [4, 8]. Interestingly, our results also displayed a specific localization of RACK1 in the cytoplasm in NPC tissues and cells. This suggests that RACK1 plays roles in NPC through regulating some pathways that require interaction of multiple proteins in the cytoplasm.

There is an interesting fact in our research; the mRNA expression level of RACK1 in NPC cells was not significantly higher than that of NP69, even in some NPC cells RACK1 mRNA expression level was lower than NP69. This is similar to some previous studies on the roles of RACK1 in breast cancer [10, 11] and thyroid cancer [31]. These studies also obtained inconsistent results; the mRNA expression level of RACK1 in tumor tissues was lower than normal tissues, whereas the protein expression level of RACK1 in tumor tissues was significantly higher than normal tissues. This implies the existence of a complicated post-transcriptional regulation of RACK1. As we know, post-transcriptional regulation is an important regulatory mechanism in nasopharyngeal carcinoma. Besides, although NP69 is an immortalized nasopharyngeal epithelial cell, it is a relatively normal cell line compared with tumor cell, so maybe it can not fully reflect the physiological characteristics of RACK1 expression in normal nasopharyngeal epithelial tissues.

Discovery of more reliable molecular biomarkers is meaningful for NPC management. Researchers have made considerable achievements in recent years. Some valuable biomarkers such as VEGF protein, IL-8 receptor A [32], Bmi-1 [32, 33], etc. have been found to be associated with the development and poor prognosis of NPC. Some important molecules such as Tiam1 [32, 33], TSLC1 [34], FGFR4 [35], FLOT1 [36] and CCL2-CCR2 axis [37], etc. are closely correlated the metastasis, invasion and EMT of NPC. Moreover, HOTAIR, a cancer-related long non-coding RNA is discovered to be up-regulated in NPC and associated with poor prognosis as well [38]. However, it is in NPC that there has been no report of the roles of RACK1, though RACK1 has been demonstrated to be a potential prognostic indicator in multiple cancers including breast cancer [10, 11, 39] and pulmonary adenocarcinomas [12, 40] and so on. In the present study, we found that RACK1 contributed to NPC cell proliferation and invasion. Silencing of RACK1 expression suppressed NPC cell proliferation and migration. RACK1 expression was significantly associated with lymph node invasion and clinical stage in NPC patients. Our observations first indicate that RACK1 can promote the NPC development and progression, suggesting RACK1 as a potential prognostic biomarker or therapeutic target for clinical applications.

It is well established that RACK1 promotes cell proliferation and invasion via its interactions with diverse partners or through various signaling pathways. RACK1 can enhance cell growth by activating Sonic hedgehog (SHH) signaling in NSCLC [41] and MKK7/JNK activity in HCC [42]. It can increase THP1 proliferation through activating GSK3β activity in acute myeloid leukemia cells [43]. It is worth noting that RACK1 has recently emerged as an essential molecule for FAK’s function as it interacts with FAK to form complex and regulates FAK activity. Phosphorylation of RACK1 on Tyr-52 by c-Abl mediates the interaction with FAK [23–25, 44]. Suppression of RACK1 expression can disrupt FAK activity and reduce cell migration. Besides, RACK1 can modulate PI3K/Akt signaling or activates Akt to propel cell proliferation and invasion/metastasis in VEGF/Flt1-mediated endothelial cells [26], mouse hepatocarcinoma cells [27], renal cell carcinoma [28], and some other cancers [29, 30]. In the present study, our results also revealed that RACK1 activated Akt and FAK in NPC. To our knowledge, PTEN is a crucial factor regulating PI3K/Akt and activating FAK. Its expression can be down-regulated by either CpG methylation at its promoter [45] or EBV-encoded miRNAs [46] in NPC. Interestingly, Gunaratne et al.’s [46] data show that RACK1 can combine with PTEN, so it is possible that RACK1 may affect cell growth and metastasis/invasion of NPC through PTEN/PI3K/Akt and FAK pathway, which we are planning to fully validate in the following studies. In addition, it has been reported that RACK1 may play a role in the virus infection and immune system. EBV encoded proteins A73, BZLF1 and ZEBRA can interact with RACK1 [19–22]. This provides clues that the interaction between RACK1 and EBV-associated immunity may be one of the mechanisms underlying NPC development and progression.

## Conclusion

The present study demonstrated that high expression of RACK1 was closely associated with cell proliferation and metastasis/invasion in NPC. Regulation of the PI3K/Akt/FAK pathway was one of functional mechanisms of RACK1 in NPC. Our findings are useful for further understanding of RACK1-based molecular mechanisms underlying NPC progression and providing a potential prognostic biomarker or therapeutic target.

## Additional files

10.1186/s12967-016-0885-x (a) Confocal analysis was used to detect the location of RACK1 in NPC tissue. Claudin-1 was used as a cell membrane marker. The nuclei were stained with DAPI. Magnification, ×600. Scale bar, 10μm. Investigated wavelength: 473nm (green); red (559nm); 405nm (blue). (b) Immunofluorescence images showed the location of RACK1 in NPC cell. Magnification, ×400. Scale bar, 10μm. Green /EX:450-490nm, EM:520nm; blue/ EX:340-380nm, EM:435-485nm.

10.1186/s12967-016-0885-x (a) NPC cells were transducted with GFP-RACK1 plasmid or GFP-Tag control plasmid. Green fluorescence show exogenous RACK1 effectively expressed in RACK1 transfected NPC cells, but non-specifically expressed in control plasmid group. Magnification, × 400. Scale bar, 25μm. (b) Western Blot shows that GFP-RACK1 is a fusion protein, about 65 kDa. (c) The phosphorylation levels of p-Akt(S473) and p-FAK(Y397) in RACK1-loss NPC cells partly restored after restitution of RACK1 with Flag-RACK1 virus. (d, e) The expression levels of EMT markers (E-cadherin, N-cadherin and Vimentin) were measured by western blot after overexpression or knockdown of RACK1 in NPC cells.

## Abbreviations

RACK1: the receptor for activated C kinase 1
NPC: nasopharyngeal carcinoma
NP: non-cancerous nasopharyngeal tissues
NP69: immortalized nasopharyngeal epithelial cell line
EMT: epithelial-mesenchymal transition
ESCC: esophageal squamous cell carcinoma
OSCC: oral squamous cell carcinoma
NSCLC: non-small-cell lung cancer
Tiam1: T lymphoma invasion and metastasis inducing factor 1
TSLC1: tumor suppressor in lung cancer 1
FGFR4: fibroblast growth factor receptor 4
FLOT1: flotillin-1
CCL2-CCR2: chemokine C-C motif ligand 2 and chemokine C-C motif receptor type 2

## References

[1] Wei WI, Sham JS (2005). Nasopharyngeal carcinoma. Lancet.

[2] Chan ATC, Teo PML, Johnson PJ (2002). Nasopharyngeal carcinoma. Ann Oncol.

[3] Lin JC (2003). Phase III study of concurrent chemoradiotherapy versus radiotherapy alone for advanced nasopharyngeal carcinoma: positive effect on overall and progression-free survival. J Clin Oncol.

[4] Li JJ, Xie D (2015). RACK1, a versatile hub in cancer. Oncogene.

[5] Ron D, Chen CH, Caldwell J, Jamieson L, Orr E, Mochly-Rosen D (1994). Cloning of an intracellular receptor for protein kinase C: a homolog of the beta subunit of G proteins. Proc Natl Acad Sci USA.

[6] Berns H, Humar R, Hengerer B, Kiefer FN, Battegay EJ (2000). RACK1 is up-regulated in angiogenesis and human carcinomas. FASEB J.

[7] McCahill A, Warwicker J, Bolger GB, Houslay MD, Yarwood SJ (2002). The RACK1 scaffold protein: a dynamic cog in cell response mechanisms. Mol Pharmacol.

[8] Adams DR, Ron D, Kiely PA (2011). RACK1, A multifaceted scaffolding protein: structure and function. Cell Commun Signal.

[9] Egidy G, Jule S, Bosse P, Bernex F, Geffrotin C, Vincent-Naulleau S, Horak V, Sastre-Garau X, Panthier JJ (2008). Transcription analysis in the MeLiM swine model identifies RACK1 as a potential marker of malignancy for human melanocytic proliferation. Mol Cancer.

[10] Al-Reefy S, Mokbel K (2010). The role of RACK1 as an independent prognostic indicator in human breast cancer. Breast Cancer Res Treat.

[11] Cao XX, Xu JD, Xu JW, Liu XL, Cheng YY, Wang WJ, Li QQ, Chen Q, Xu ZD, Liu XP (2010). RACK1 promotes breast carcinoma proliferation and invasion/metastasis in vitro and in vivo. Breast Cancer Res Treat.

[12] Zhong X, Li M, Nie B, Wu F, Zhang L, Wang E, Han Y (2013). Overexpressions of RACK1 and CD147 associated with poor prognosis in stage T1 pulmonary adenocarcinoma. Ann Surg Oncol.

[13] Ruan Y, Sun L, Hao Y, Wang L, Xu J, Zhang W, Xie J, Guo L, Zhou L, Yun X (2012). Ribosomal RACK1 promotes chemoresistance and growth in human hepatocellular carcinoma. J Clin Invest.

[14] Hu F, Tao Z, Wang M, Li G, Zhang Y, Zhong H, Xiao H, Xie X, Ju M (2013). RACK1 promoted the growth and migration of the cancer cells in the progression of esophageal squamous cell carcinoma. Tumour Biol.

[15] Wang Z, Zhang B, Jiang L, Zeng X, Chen Y, Feng X, Guo Y, Chen Q (2009). RACK1, an excellent predictor for poor clinical outcome in oral squamous carcinoma, similar to Ki67. Eur J Cancer.

[16] Chang BY, Conroy KB, Machleder EM, Cartwright CA (1998). RACK1, a receptor for activated C kinase and a homolog of the beta subunit of G proteins, inhibits activity of src tyrosine kinases and growth of NIH 3T3 cells. Mol Cell Biol.

[17] Bjorndal B, Myklebust LM, Rosendal KR, Myromslien FD, Lorens JB, Nolan G, Bruland O, Lillehaug JR (2007). RACK1 regulates Ki-Ras-mediated signaling and morphological transformation of NIH 3T3 cells. Int J Cancer.

[18] Deng YZ, Yao F, Li JJ, Mao ZF, Hu PT, Long LY, Li G, Ji XD, Shi S, Guan DX (2012). RACK1 suppresses gastric tumorigenesis by stabilizing the beta-catenin destruction complex. Gastroenterology.

[19] Al-Mozaini M, Bodelon G, Karstegl CE, Jin B, Al-Ahdal M, Farrell PJ (2009). Epstein-Barr virus BART gene expression. J Gen Virol.

[20] Lo AK, Liu Y, Wang X, Wong YC, Kai FLC, Huang DP, Tsao SW (2001). Identification of downstream target genes of latent membrane protein 1 in nasopharyngeal carcinoma cells by suppression subtractive hybridization. Biochim Biophys Acta.

[21] Baumann M, Gires O, Kolch W, Mischak H, Zeidler R, Pich D, Hammerschmidt W (2000). The PKC targeting protein RACK1 interacts with the Epstein-Barr virus activator protein BZLF1. Eur J Biochem.

[22] Tardif M, Savard M, Flamand L, Gosselin J (2002). Impaired protein kinase C activation/translocation in Epstein–Barr virus-infected monocytes. J Biol Chem.

[23] Serrels B, Sandilands E, Serrels A, Baillie G, Houslay MD, Brunton VG, Canel M, Machesky LM, Anderson KI, Frame MC (2010). A complex between FAK, RACK1, and PDE4D5 controls spreading initiation and cancer cell polarity. Curr Biol.

[24] Dave JM, Kang H, Abbey CA, Maxwell SA, Bayless KJ (2013). Proteomic profiling of endothelial invasion revealed receptor for activated C kinase 1 (RACK1) complexed with vimentin to regulate focal adhesion kinase (FAK). J Biol Chem.

[25] Kiely PA, Baillie GS, Barrett R, Buckley DA, Adams DR, Houslay MD, O’Connor R (2009). Phosphorylation of RACK1 on tyrosine 52 by c-Abl is required for insulin-like growth factor I-mediated regulation of focal adhesion kinase. J Biol Chem.

[26] Wang F, Yamauchi M, Muramatsu M, Osawa T, Tsuchida R, Shibuya M (2011). RACK1 regulates VEGF/Flt1-mediated cell migration via activation of a PI3 K/Akt pathway. J Biol Chem.

[27] Wu J, Meng J, Du Y, Huang Y, Jin Y, Zhang J, Wang B, Zhang Y, Sun M, Tang J (2013). RACK1 promotes the proliferation, migration and invasion capacity of mouse hepatocellular carcinoma cell line in vitro probably by PI3K/Rac1 signaling pathway. Biomed Pharmacother.

[28] He X, Wang J, Messing EM, Wu G (2011). Regulation of receptor for activated C kinase 1 protein by the von Hippel–Lindau tumor suppressor in IGF-I-induced renal carcinoma cell invasiveness. Oncogene.

[29] Li G, Ji XD, Gao H, Zhao JS, Xu JF, Sun ZJ, Deng YZ, Shi S, Feng YX, Zhu YQ (2012). EphB3 suppresses non-small-cell lung cancer metastasis via a PP2A/RACK1/Akt signalling complex. Nat Commun.

[30] Lin Y, Cui M, Teng H, Wang F, Yu W, Xu T (2014). Silencing the receptor of activated C-kinase 1 (RACK1) suppresses tumorigenicity in epithelial ovarian cancer in vitro and in vivo. Int J Oncol.

[31] Myklebust LM, Akslen LA, Varhaug JE, Lillehaug JR (2011). Receptor for activated protein C kinase 1 (RACK1) is overexpressed in papillary thyroid carcinoma. Thyroid.

[32] Cho WC (2007). Nasopharyngeal carcinoma: molecular biomarker discovery and progress. Mol Cancer.

[33] Song LB (2006). Bmi-1 Is a novel molecular marker of nasopharyngeal carcinoma progression and immortalizes primary human nasopharyngeal epithelial cells. Cancer Res.

[34] Lung HL, Leung Cheung AK, Xie D, Cheng Y, Kwong FM, Murakami Y, Guan XY, Sham JS, Chua D, Protopopov AI (2006). TSLC1 is a tumor suppressor gene associated with metastasis in nasopharyngeal carcinoma. Cancer Res.

[35] Shi S, Li X, You B, Shan Y, Cao X, You Y (2015). High expression of FGFR4 enhances tumor growth and metastasis in nasopharyngeal carcinoma. J Cancer.

[36] Cao S, Cui Y, Xiao H, Mai M, Wang C, Xie S, Yang J, Wu S, Li J, Song L (2016). Upregulation of flotillin-1 promotes invasion and metastasis by activating TGF-beta signaling in nasopharyngeal carcinoma. Oncotarget.

[37] Yang J, Lv X, Chen J, Xie C, Xia W, Jiang C, Zeng T, Ye Y, Ke L, Yu Y (2015). CCL2-CCR2 axis promotes metastasis of nasopharyngeal carcinoma by activating ERK1/2-MMP2/9 pathway. Oncotarget.

[38] Nie Y, Liu X, Qu S, Song E, Zou H, Gong C (2013). Long non-coding RNA HOTAIR is an independent prognostic marker for nasopharyngeal carcinoma progression and survival. Cancer Sci.

[39] Cao XX, Xu JD, Liu XL, Xu JW, Wang WJ, Li QQ, Chen Q, Xu ZD, Liu XP (2010). RACK1: a superior independent predictor for poor clinical outcome in breast cancer. Int J Cancer.

[40] Nagashio R, Sato Y, Matsumoto T, Kageyama T, Satoh Y, Shinichiro R, Masuda N, Goshima N, Jiang SX, Okayasu I (2010). Expression of RACK1 is a novel biomarker in pulmonary adenocarcinomas. Lung Cancer.

[41] Shi S, Deng YZ, Zhao JS, Ji XD, Shi J, Feng YX, Li G, Li JJ, Zhu D, Koeffler HP (2012). RACK1 promotes non-small-cell lung cancer tumorigenicity through activating sonic hedgehog signaling pathway. J Biol Chem.

[42] Guo Y, Wang W, Wang J, Feng J, Wang Q, Jin J, Lv M, Li X, Li Y, Ma Y (2013). Receptor for activated C kinase 1 promotes hepatocellular carcinoma growth by enhancing mitogen-activated protein kinase kinase 7 activity. Hepatology.

[43] Zhang D, Wang Q, Zhu T, Cao J, Zhang X, Wang J, Wang X, Li Y, Shen B, Zhang J (2013). RACK1 promotes the proliferation of THP1 acute myeloid leukemia cells. Mol Cell Biochem.

[44] Serrels B, Sandilands E, Frame MC (2011). Signaling of the direction-sensing FAK/RACK1/PDE4D5 complex to the small GTPase Rap1. Small GTPases.

[45] Li J, Gong P, Lyu X, Yao K, Li X, Peng H (2014). Aberrant CpG island methylation of PTEN is an early event in nasopharyngeal carcinoma and a potential diagnostic biomarker. Oncol Rep.

[46] Gunaratne J, Goh MX, Swa HL, Lee FY, Sanford E, Wong LM, Hogue KA, Blackstock WP, Okumura K (2011). Protein interactions of phosphatase and tensin homologue (PTEN) and its cancer-associated G20E mutant compared by using stable isotope labeling by amino acids in cell culture-based parallel affinity purification. J Biol Chem.

