# Supplementary figures and images for: The important role of the receptor for activated C kinase 1 (RACK1) in nasopharyngeal carcinoma progression
Source: J Transl Med. 2016 May 11;14:131. doi: 10.1186/s12967-016-0885-x (PMC4864934; doi:10.1186/s12967-016-0885-x)

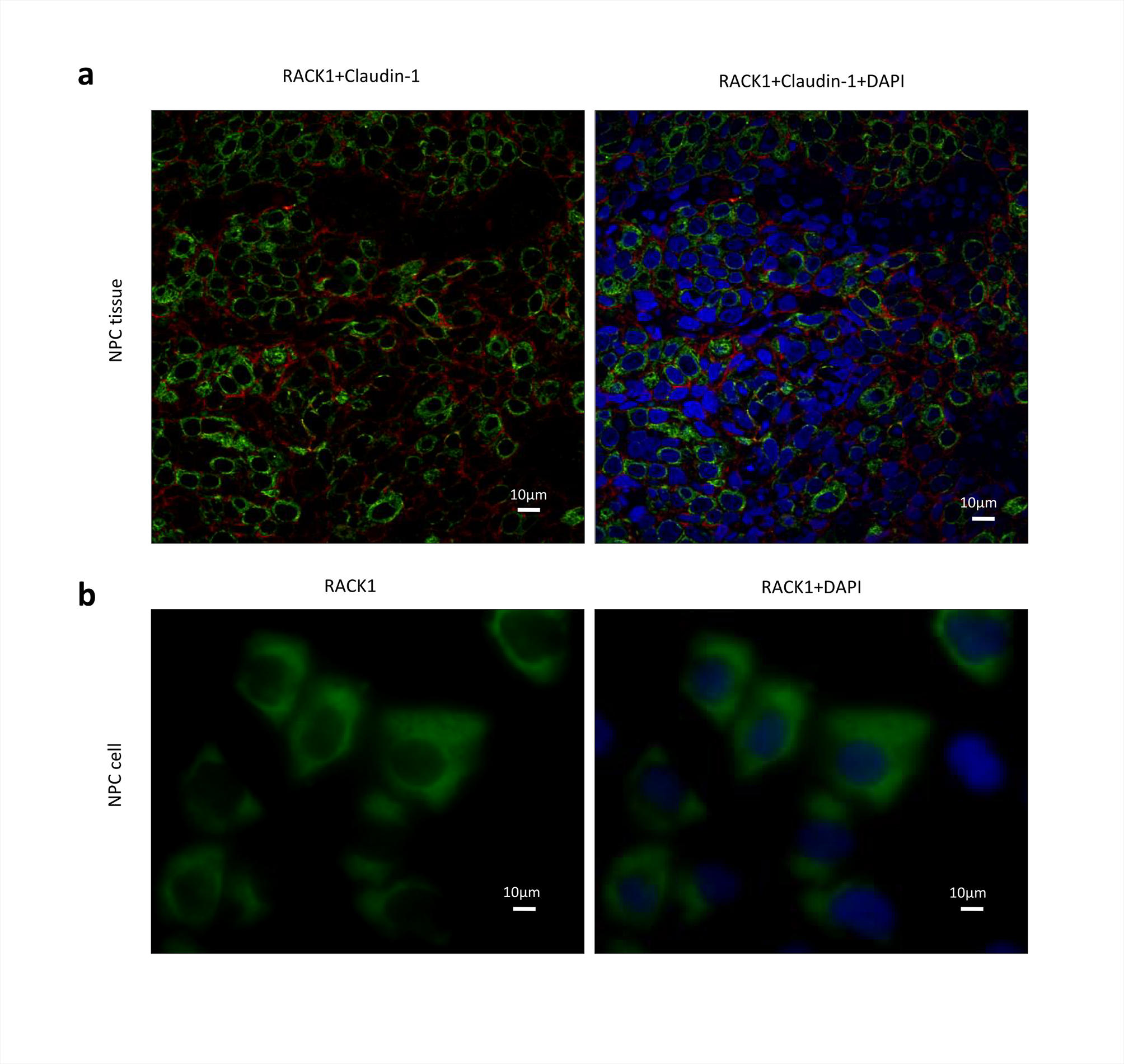

Supplement: Supplementary file 1 — 10.1186/s12967-016-0885-x (a) Confocal analysis was used to detect the location of RACK1 in NPC tissue. Claudin-1 was used as a cell membrane marker. The nuclei were stained with DAPI. Magnification, ×600. Scale bar, 10μm. Investigated wavelength: 473nm (green); red (559nm); 405nm (blue). (b) Immunofluorescence images showed the location of RACK1 in NPC cell. Magnification, ×400. Scale bar, 10μm. Green /EX:450-490nm, EM:520nm; blue/ EX:340-380nm, EM:435-485nm. [file 12967_2016_885_MOESM1_ESM.tif]

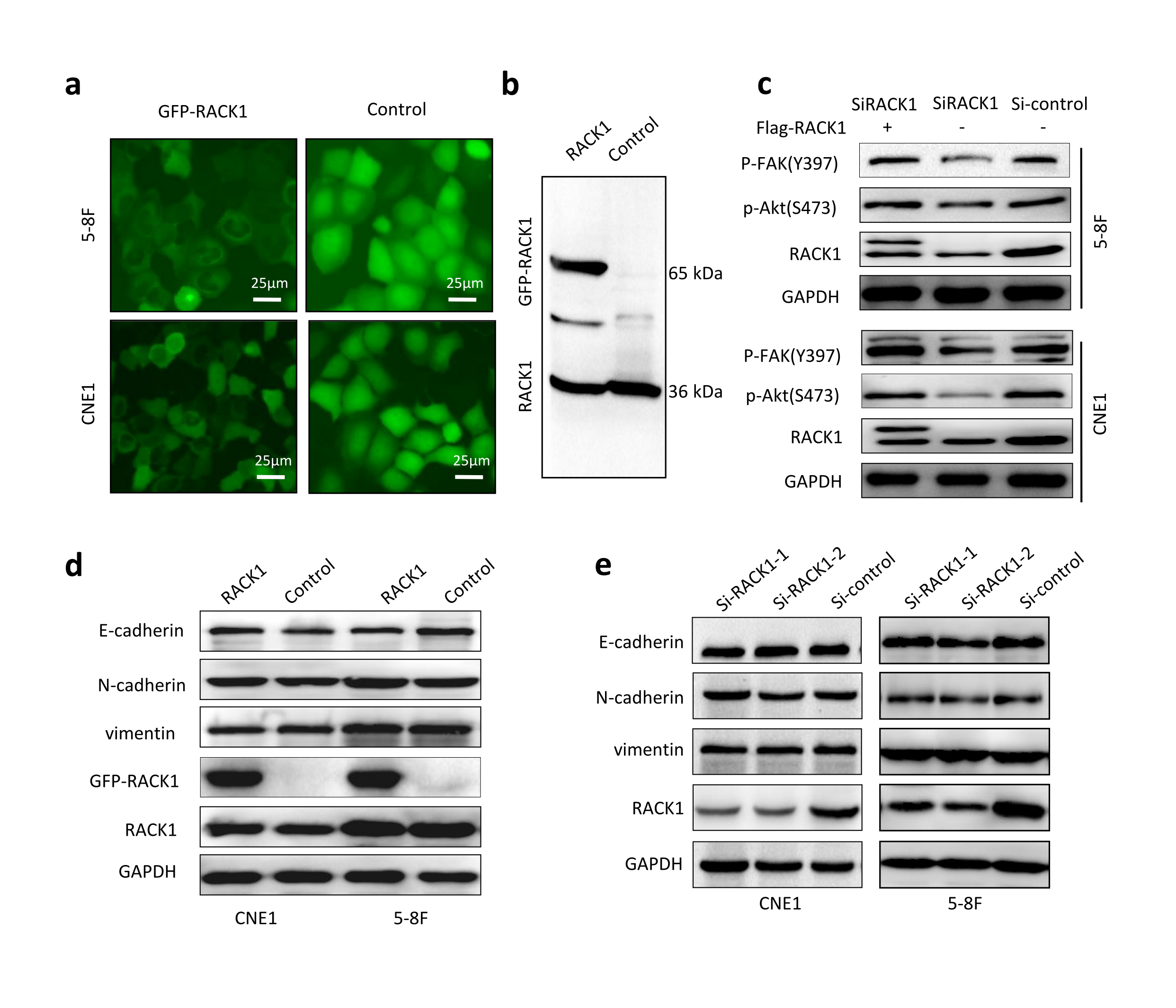

Supplement: Supplementary file 2 — 10.1186/s12967-016-0885-x (a) NPC cells were transducted with GFP-RACK1 plasmid or GFP-Tag control plasmid. Green fluorescence show exogenous RACK1 effectively expressed in RACK1 transfected NPC cells, but non-specifically expressed in control plasmid group. Magnification, × 400. Scale bar, 25μm. (b) Western Blot shows that GFP-RACK1 is a fusion protein, about 65 kDa. (c) The phosphorylation levels of p-Akt(S473) and p-FAK(Y397) in RACK1-loss NPC cells partly restored after restitution of RACK1 with Flag-RACK1 virus. (d, e) The expression levels of EMT markers (E-cadherin, N-cadherin and Vimentin) were measured by western blot after overexpression or knockdown of RACK1 in NPC cells. [file 12967_2016_885_MOESM2_ESM.tif]
